# Extraction and Purification of Polysaccharides from Thermotolerant *Pyropia haitanensis* Strain SW-81 and Its Hypolipidemic Effects on Oleic Acid-Induced Lipid Accumulation in HepG2 Cells

**DOI:** 10.3390/md24070241

**Published:** 2026-07-08

**Authors:** Jiawei Zhong, Hongchang Ding, Jogeir Toppe, Kaiyue Chen, Menghan Wei, Xin Chen, Long Zhang, Quancai Sun, Ye Peng, Wenhui Wu, Wanqiang Wu, Xichang Wang

**Affiliations:** 1 College of Food Science and Technology, Shanghai Ocean University, Shanghai 201306, China; jiaweizhong1215@163.com (J.Z.); 18638709512@163.com (K.C.); 13523299021@163.com (M.W.); chenxin20002021@163.com (X.C.); l-zhang@shou.edu.cn (L.Z.); 2 Key Laboratory of Exploration and Utilization of Aquatic Genetic Resources, Ministry of Education, Shanghai Ocean University, Shanghai 201306, China; hcding@shou.edu.cn; 3 Shanghai Engineering Research Center of Aquaculture, Shanghai Ocean University, Shanghai 201306, China; 4 Food and Agriculture Organization of the United Nations (FAO), 00153 Rome, Italy; jogeir.toppe@fao.org; 5 Shanghai Engineering Research Center of Aquatic-Product Processing and Preservation, Shanghai 201306, China; 6 Faculty of Medicine, Macau University of Science and Technology, Taipa, Macao 999078, China; qcsun@must.edu.mo (Q.S.); pengye@must.edu.mo (Y.P.); 7 Department of Marine Pharmacology, College of Food Science and Technology, Shanghai Ocean University, Shanghai 201306, China; whwu@shou.edu.cn; 8 Putuo Sub-Center of International Joint Research Center for Marine Biological Sciences, Zhoushan 316104, China; 9 Marine Biomedical Science and Technology Innovation Platform of Lin-Gang Special Area, Shanghai 201306, China; 10 International Research Center for Food and Health, College of Food Science and Technology, Shanghai Ocean University, Shanghai 201306, China

**Keywords:** ultrasound-assisted enzyme extraction, response surface methodology, *Pyropia haitanensis*, polysaccharides, hypolipidemic activity

## Abstract

*Pyropia haitanensis* polysaccharides have attracted growing attention for their diverse biological activities. In this study, we developed a synergistic extraction approach combining ultrasonic-assisted treatment and enzymatic hydrolysis using cellulase and pectinase. Response surface methodology (RSM) was applied to optimize the extraction conditions, which were determined as follows: 1.48% cellulase, 1.47% pectinase, 180 W ultrasonic power, and 65.9 °C temperature. Under these conditions, the polysaccharide yield reached 10.184 ± 0.27%. The crude extract was then purified through sequential DEAE Sepharose FastFlow and Sephadex G-75 chromatography, resulting in the purified fraction PPHP3. Monosaccharide analysis revealed that galactose, glucose, and glucuronic acid constituted the primary components in a molar ratio of 98.3:0.46:1.24. This polysaccharide exhibited a weight-average molecular weight of 25.208 kDa, a sulfate content of 8.64 ± 0.05%. In hypolipidemic assays using oleic acid-induced HepG2 cells, PPHP3 significantly reduced intracellular triglycerides (TG), total cholesterol (TC), and low-density lipoprotein cholesterol (LDL-C), while simultaneously increasing HDL-C levels. These findings highlight the potential of *P. haitanensis* polysaccharides for hypolipidemic applications and establish a scientific foundation for their development in therapeutic and practical contexts.

## 1. Introduction

Rapid socio-economic development and improved living standards have transformed dietary patterns and lifestyles, with rising consumption of high-energy, high-fat diets and declining physical activity. This shift has fueled a growing epidemic of lipid metabolism disorders [1,2]. Dyslipidemia, a core component of metabolic syndrome, is characterized by abnormal serum lipid and lipoprotein levels, manifesting as elevated total cholesterol (TC), triglycerides (TG), and low-density lipoprotein cholesterol (LDL-C), alongside reduced high-density lipoprotein cholesterol (HDL-C)—an endogenous protector against cardiovascular disease. As a major independent risk factor for severe chronic conditions including atherosclerosis, non-alcoholic fatty liver disease, and cardio-cerebrovascular diseases, dyslipidemia has emerged as a significant global health threat [3,4]. Epidemiological data reveals that over 40% of Chinese adults suffer from dyslipidemia, with increasing incidence among younger populations—a trend exacerbating the disease burden [5].

At present, lipid-lowering therapy primarily relies on statins and fibrates. While these chemical agents exhibit robust efficacy, long-term administration may induce adverse reactions, including hepatotoxicity, nephrotoxicity, myotoxicity, and new-onset diabetes. Ongoing safety concerns and patient tolerability issues associated with these medications highlight the need for safer alternatives [6,7]. Consequently, the discovery and development of high-efficacy, low-toxicity lipid-lowering bioactives or nutraceuticals from natural sources has become a focal area in food science and preventive medicine research.

To align with the “Great Food Concept” and food security objectives, China is advancing the construction of a “Blue Granary,” through transitioning marine fisheries from coastal aquaculture to offshore intelligent cultivation systems. As guided by this national strategy, the industry is evolving from traditional “raw material production” toward an integrated value chain emphasizing “technological innovation” and “high-value product development.” As a pivotal component of modern marine ranching, seaweed cultivation not only directly supplies food but also serves as a rich reservoir of bioactive compounds, including polysaccharides, proteins, and polyphenols, with significant potential for functional food and pharmaceutical applications.

Algal polysaccharides, as essential biomacromolecules ubiquitously found in marine organisms, have been extensively investigated for their versatile bioactivities, including antioxidant, lipid-lowering, and anti-inflammatory properties. Notably, Koh HSA et al. [8] demonstrated that low-molecular-weight fucoidan exhibited superior secondary antioxidant capacity compared to the synthetic antioxidant butylated hydroxyanisole (BHA). Li et al. [9] isolated two polysaccharide fractions from *Ulva pertusa*, both fractions displayed significantly enhanced hypolipidemic activity compared to the crude polysaccharide extract. Gong et al. [10] elucidated that the PHPD-IV-4 polysaccharide from *Porphyra haitanensis* activated macrophage immunoregulation through modulation of MAPK signaling molecule phosphorylation. Among marine algae, *Pyropia haitanensis* is an economically vital and widely cultivated species along the southeastern coast of China. Beyond its traditional use as an edible and medicinal resource, this alga represents a pivotal source of marine bioactive polysaccharides. Existing studies have substantiated the immunomodulatory and antitumor properties of its polysaccharide components, positioning them as promising candidates for functional food applications.

Notwithstanding these advancements, investigations into the lipid-lowering bioactivity of *Pyropia haitanensis* polysaccharides are hindered by significant limitations. Firstly, extant research predominantly focuses on crude polysaccharides or mixed fractions extracted via traditional hot water methods, lacking a systematic dissection of the structure–activity relationship (SAR) governing the hypolipidemic efficacy of specific homogeneous polysaccharide fractions. For instance, Dong et al. [11] reported that hot water-extracted polysaccharides from *P. haitanensis* primarily comprise galactose, glucose, and fucose; however, the bioactivity and structural features of their purified fractions remain elusive. Secondly, the dense cell wall structure of *P. haitanensis* poses a challenge for extraction efficiency. While single-step hot water or enzymatic extraction methods suffer from limited yield and potential damage to active structures [12,13], advanced techniques face distinct drawbacks: microwave-assisted extraction may induce polysaccharide degradation despite higher rates [14], while subcritical water extraction requires costly equipment and complex operational procedures [15]. Consequently, developing efficient and gentle cell disruption strategies to maximize the liberation of bioactive polysaccharides remains a critical technical impediment hindering their high-value utilization. Ultrasonic extraction is a green, efficient auxiliary extraction technology widely used in the preparation of marine algal polysaccharides. Its core advantage lies in the cavitation effect, mechanical shearing and thermal agitation generated by ultrasonic waves, which can rapidly break the compact cell wall of algae and accelerate the dissolution of polysaccharides, significantly shortening the extraction time and improving the extraction efficiency [16]. However, single ultrasonic extraction has obvious drawbacks: excessive ultrasonic power and prolonged action time will cause thermal degradation and chain scission of polysaccharides, destroying their structural integrity and biological activity; in addition, single ultrasonic extraction has limited ability to degrade the cellulose–pectin complex in algal cell walls, resulting in insufficient polysaccharide release. Ultrasonic-assisted enzymatic extraction combines the mild catalytic effect of enzyme and the physical disruption effect of ultrasound, which overcomes the shortcomings of single extraction methods. Enzymes (cellulase, pectinase) can specifically degrade the structural components of algal cell walls without damaging the polysaccharide structure [17], and ultrasound can enhance the mass transfer efficiency and enzyme catalytic efficiency, so as to achieve high yield and mild extraction of polysaccharides, which has become an optimal strategy for the extraction of marine algal bioactive polysaccharides.

In response to these challenges, Professor Ding’s laboratory at the College of Fisheries and Life Sciences, Shanghai Ocean University, developed a novel *P. haitanensis* strain SW-81 through ultraviolet irradiation of wild-type *P. haitanensis* gametophytic blades followed by heat stress acclimation. Notably, SW-81 not only retains the high-yield and thermotolerance traits of SF-2 but also demonstrates accelerated differentiation upon filamentous inoculation onto shell substrates [18]. To address this, we employed an ultrasonic-assisted enzymatic extraction protocol to isolate polysaccharides from SW-81. Utilizing response surface methodology (RSM) based on Box–Behnken design, we systematically optimized key parameters (ultrasonic temperature, power, cellulase/pectinase dosages) to maximize polysaccharide yield. The extract was then fractionated via sequential chromatography on DEAE-cellulose and Sephadex G-75 columns, yielding a homogeneous polysaccharide designated PPHP3. To analyze the basic properties of PPHP3, we employed high-performance gel permeation chromatography (HPGPC) and ion chromatography (IC) to determine its molecular weight, monosaccharide composition, and basic compositional features. Additionally, an oleic acid-induced HepG2 model with lipid accumulation was developed to evaluate its lipid metabolic effects, thereby providing scientific underpinnings for *P. haitanensis* polysaccharide exploitation.

## 2. Results

### 2.1. Single-Factor Experiments

**Figure 1:**
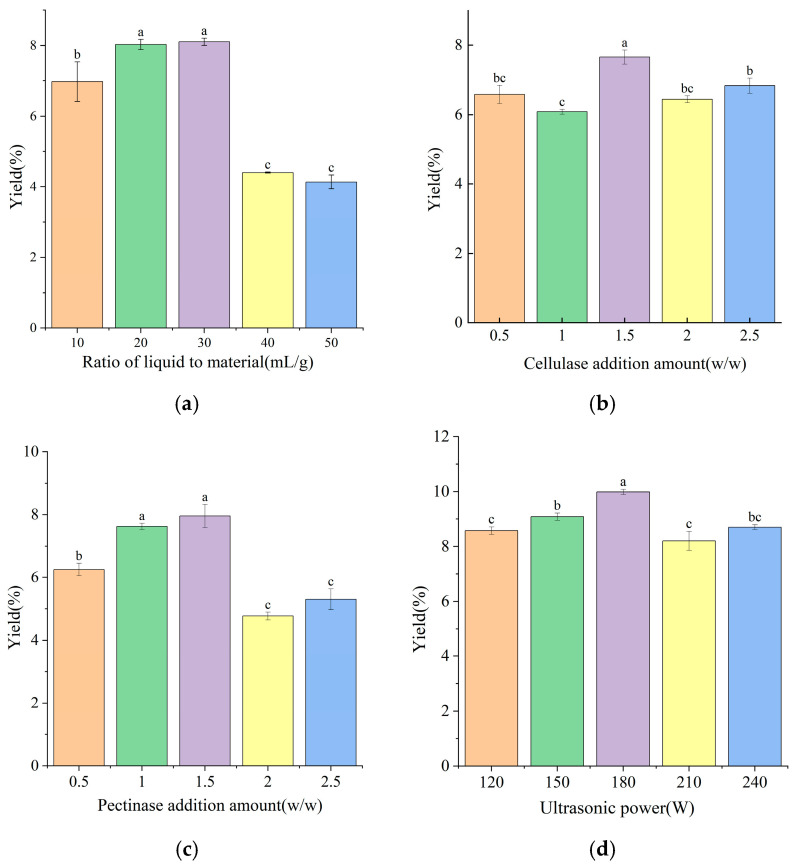

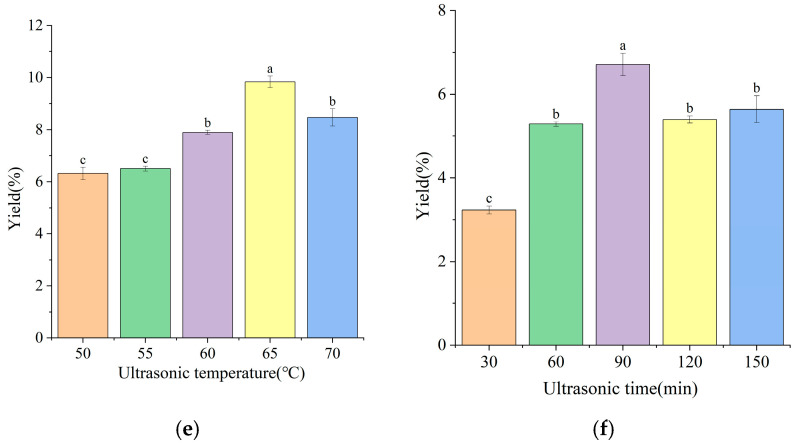
Effects of single factors on polysaccharide yield: (**a**) ratio of liquid to material; (**b**) cellulase addition amount; (**c**) pectinase addition amount, (**d**) ultrasonic power; (**e**) ultrasonic temperature; (**f**) ultrasonic time. Data are presented as mean ± SD from at least 3 independent experiments (3 replicates); Different letters indicate significant differences (*p* < 0.05).

As depicted in Figure 1a, the total sugar content of *P. haitanensis* polysaccharides exhibited a significant increase as the liquid–solid ratio rose from 10 to 30 mL/g, peaking at 8.11 ± 0.10% at a 30 mL/g ratio before gradually declining. This trend is attributed to enhanced mass transfer driven by the increased concentration gradient between intracellular and extracellular environments, which facilitates solvent penetration and polysaccharide dissolution. Conversely, excessive solvent volumes (beyond 30 mL/g) dilute the substrate concentration, impairing polysaccharide extraction efficiency and leading to diminished yields [19]. As shown in Figure 1b,c, cellulase and pectinase dosages exhibit distinct effects on *P. haitanensis* polysaccharide extraction. Both enzymes sequentially target cellulose and pectin within the cell wall, disrupting its structural integrity to promote polysaccharide liberation. Experimental results revealed that the extraction efficiency peaked at 1.5% (*w*/*w*) dosage for both cellulase and pectinase. Conversely, exceeding this threshold triggered a notable yield reduction. This trend is ascribed to excessive enzymatic activity, which promotes indiscriminate hydrolysis of polysaccharide glycosidic bonds, thereby impairing the recovery of intact target polysaccharides [20].

As depicted in Figure 1d, the polysaccharide yield initially increases significantly with rising ultrasonic power, reaching a maximum of 9.99 ± 0.09% at 180 W, before exhibiting a decline. This trend is ascribed to intensified cavitation effects, thermal agitation, and mechanical shearing at moderate power levels, which enhance solvent penetration and disrupt cell walls. Conversely, excessive ultrasonic power (beyond 180 W) subjects dissolved polysaccharides to prolonged irradiation, triggering thermal degradation and chain scission. Similarly, Figure 1e illustrates that total sugar content elevates with increasing temperature, peaking at 9.84 ± 0.22% at 65 °C. Elevated temperatures facilitate cell membrane fluidity and polysaccharide diffusion; however, temperatures exceeding 65 °C induce enzyme inactivation and thermal decomposition of polysaccharides, leading to diminished yields [21,22]. Figure 1f illustrates that the total sugar content initially escalates with prolonged ultrasonic treatment, peaking at 90 min. This trend is attributable to the cumulative thermal and mechanical effects generated over time, which disrupt cell walls and facilitate polysaccharide dissolution. However, extended extraction times (beyond 90 min) lead to diminished yields, as elevated polysaccharide concentrations in the solvent reduce osmotic driving force, hindering further liberation. Additionally, prolonged ultrasonic exposure subjects dissolved polysaccharides to prolonged shear stress and thermal degradation, accelerating chain fragmentation. It is worth clarifying that prior to conducting the response surface optimization experiments, we systematically investigated the effect of extraction time on polysaccharide yield through single-factor tests. The experimental results demonstrated that the polysaccharide yield increased remarkably with extended treatment time within the range of 30–90 min and reached its maximum at 90 min. Further prolonging the extraction time would instead decrease the yield. This is because excessive ultrasonic exposure induces thermal degradation and molecular chain breakage of the extracted polysaccharides, while also resulting in energy waste and lowered extraction efficiency [23]. Based on this, we selected 90 min as the fixed optimal extraction time for the subsequent response surface optimization.

### 2.2. Optimization of Extraction Conditions Based on Response Surface Methodology

#### Statistical Analysis and Model Fitting

Based on the Box–Behnken design, 29 experiments (Table 1) were conducted in a randomized order, presenting the designed parameters and corresponding outcomes. Through multivariate regression analysis of the experimental data, a quadratic polynomial model was developed to predict the total sugar yield of *P. haitanensis* polysaccharides, as expressed by the following equation:

Y(%) = 10.30 − 0.1021 × A − 0.1388 × B + 0.0096 × C + 0.3933 × D + 0.1630 × AB − 0.0292 × AC + 0.0185 × AD + 0.0567 × BC − 0.5368 × CD − 1.35 × A^2^ − 0.9134 × B^2^ − 1.14 × C^2^ − 1.11 × D^2^ (1)

where Y represents the polysaccharide extraction yield, and A, B, C, and D correspond to cellulase dosage, pectinase dosage, ultrasonic power, and ultrasonic temperature, respectively.

Table 2 systematically collates the variance analysis (ANOVA) findings corresponding to the quadratic response surface regression model. With a low *p*-value (*p* < 0.0001) and a high F-value (15.99), the regression model was demonstrated to be statistically highly significant [24]. Additionally, the lack-of-fit term exhibited an F-value of 0.9773 and a *p*-value of 0.5589, which indicates that the lack-of-fit effect is not statistically significant when compared with the pure error. The high R^2^ value (0.9411) and adjusted R^2^ adj value (0.8823) of the model indicate a high correlation between the experimental values and predicted values. In addition, the low coefficient of variation (4.01%) indicates high accuracy and reliability of the experimental data [25].

Table 2 elucidates the factorial impacts on *P. haitanensis* polysaccharide extraction yield. Notably, among the linear effects, ultrasonic temperature (D) demonstrates a highly significant influence (*p* < 0.01), while cellulase addition (A), pectinase addition (B), and ultrasonic power (C) exhibit nonsignificant effects (*p* > 0.05). Furthermore, the interaction between ultrasonic power and temperature (CD) reaches an extremely significant level (*p* < 0.01), contrasting with the insignificance of other interactions (AB, AC, AD, BC, BD; *p* > 0.05). Crucially, all quadratic terms (A^2^, B^2^, C^2^, D^2^) display overwhelming significance (*p* < 0.0001), indicating nonlinear relationships between factors and the extraction yield. These findings highlight temperature’s paramount role and the synergistic impact of power–temperature coupling in optimizing extraction conditions.

**Figure 2:**
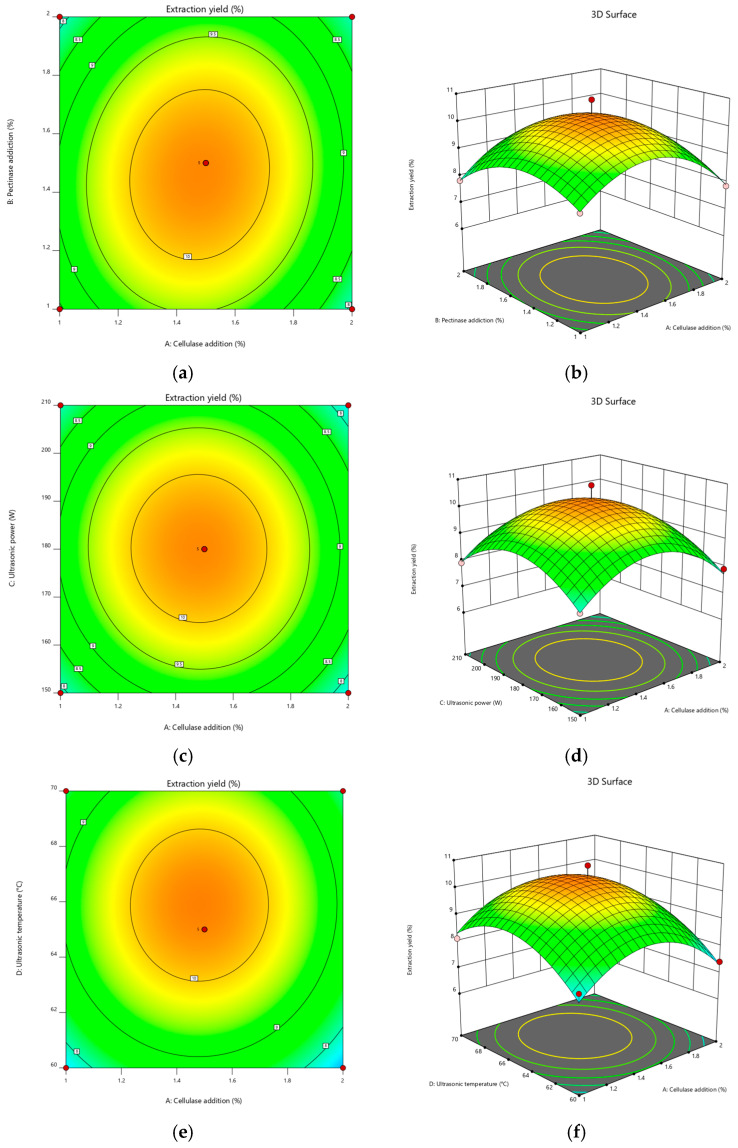

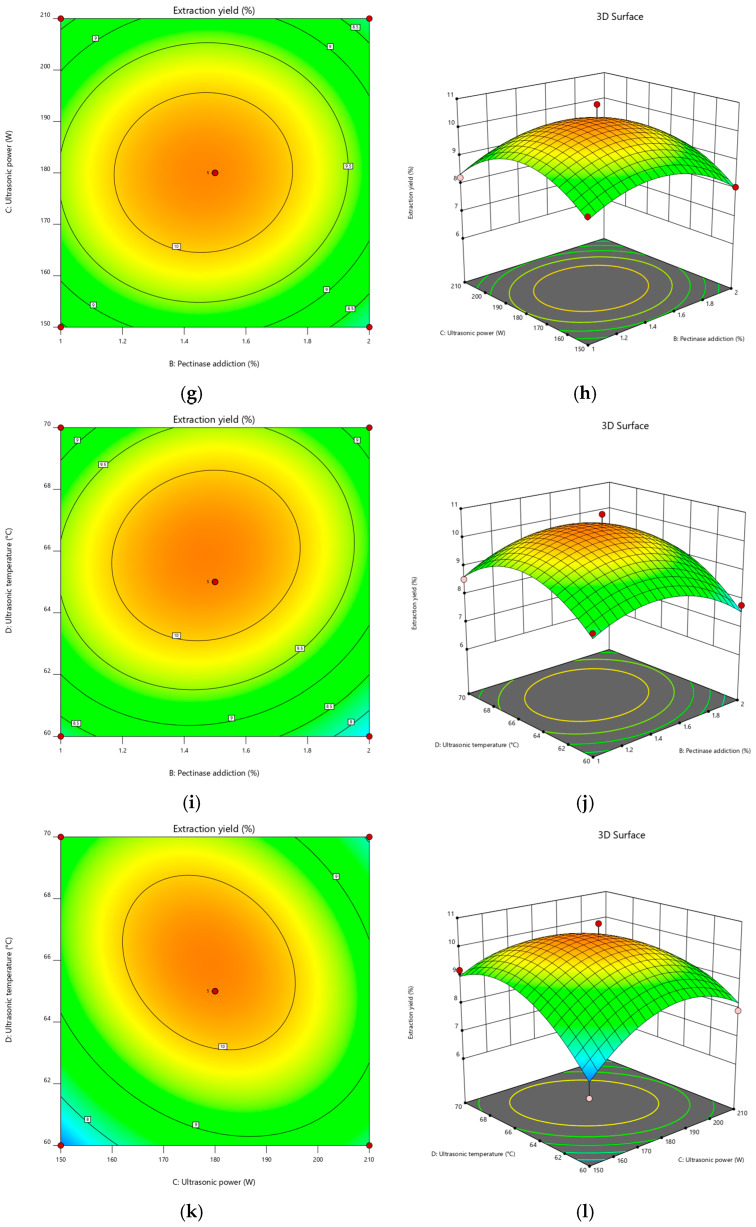
Two-dimensional contour plots (**a**,**c**,**e**,**g**,**i**,**k**) and three-dimensional response surface plots (**b**,**d**,**f**,**h**,**j**,**l**) showing the effect of cellulase addition (A), pectinase addition (B), ultrasonic power (C) and ultrasonic temperature (D) on extraction yield (Y %).

The results highlight the dominance of quadratic effects across all factors on extraction yield, followed by the linear influence of ultrasonic temperature and the interactive effect between ultrasonic power and temperature. Figure 2 vividly illustrates these interactions through 3D response surfaces and contour plots. Notably, the power–temperature interaction exhibits the steepest curvature and elliptical contours, visually validating its significance. Collectively, these diagrams reveal that within a specific range, increasing individual factors initially elevates the extraction yield, which subsequently declines in a parabolic trend—a clear indication of optimal parameter thresholds.

Through systematic analysis of numerical and graphical data, the study determined the optimal extraction parameters for *P. haitanensis* polysaccharides: a cellulase dosage of 1.48%, pectinase dosage of 1.47%, ultrasonic power of 180 W, and ultrasonic temperature of 65.9 °C. Under these conditions, the model projected a maximum extraction rate of 10.342%. To validate the model’s accuracy, three independent verification experiments were conducted. The average measured extraction rate was 10.184 ± 0.27%, aligning closely with the predicted value and showing no statistical significance (*p* > 0.05). This outcome confirms the model’s reliability in forecasting extraction efficiency.

### 2.3. Isolation and Purification of PPHP3

**Figure 3:**
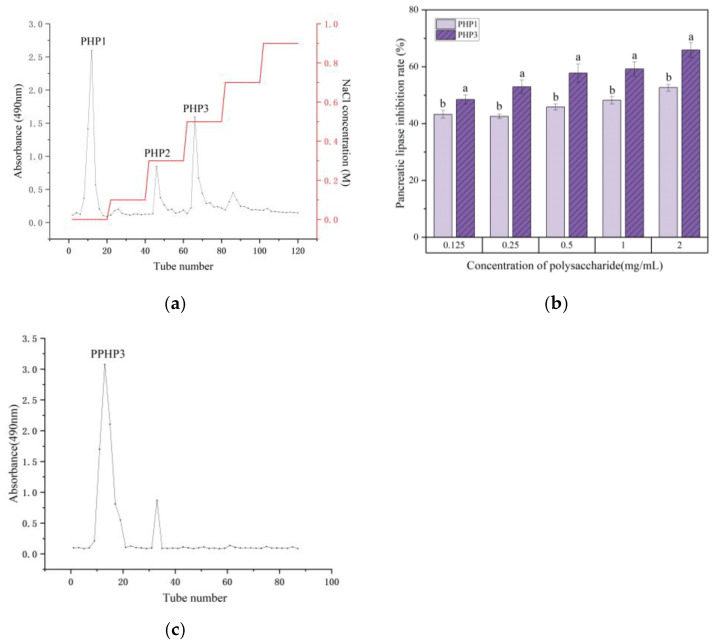
Elution curves of polysaccharide fractions. (**a**) DEAE Sepharose FastFlow column chromatography; (**b**) Pancreatic lipase inhibition rates of PHP1 and PHP3. Data are presented as mean ± standard deviation (SD). Different letters above bars within the same concentration indicate a significant difference between PHP1 and PHP3 (*p* < 0.05); (**c**) Sephadex G-75 column chromatography for PPHP3 purification.

Three polysaccharide sub-fractions, namely PHP1, PHP2 and PHP3, were isolated and collected based on the elution profile of DEAE Sepharose Fast Flow column chromatography (Figure 3a). The yields of PHP1, PHP2 and PHP3 were determined to be 46.47%, 15.09% and 28.53%, respectively. We further evaluated their in vitro pancreatic lipase inhibition activities to screen the target fraction. The results in Figure 3b showed that PHP3 exhibited the highest inhibitory activity, with an inhibition rate of 65.88 ± 2.61% at the concentration of 2 mg/mL, which was significantly higher than that of PHP1 (52.64 ± 1.18%, *p* < 0.05). PHP2 was discarded owing to its excessively low yield, which rendered sufficient sample collection unfeasible. By contrast, PHP3 displayed both satisfactory yield and favorable bioactivity; accordingly, this fraction was selected for subsequent further purification, structural characterization and in vitro cell-based assays. The PHP3 fraction was first desalted and subsequently further purified via Sephadex G-75 column chromatography, ultimately yielding the purified polysaccharide component PPHP3 (Figure 3c).

### 2.4. Chemical Composition Analysis of PPHP3

**Figure 4:**
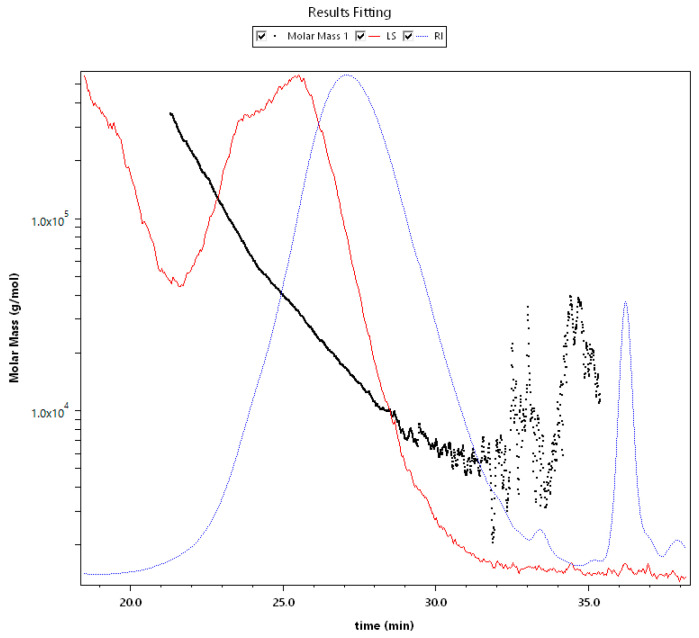
HPGPC elution profile of PPHP3 (Black dots represent real-time molar mass; red line = light scattering (LS) signal; blue dotted line = refractive index (RI) signal).

According to the determination results of the phenol–sulfuric acid method, as showned in Table 3, the polysaccharide content of PPHP3 was 84.34 ± 2.25%. The protein and sulfate contents in PPHP3 were 0.89 ± 0.05% and 8.64 ± 0.05%, respectively. HPGPC results showed that the weight-average molecular weight of PPHP3 was 25.208 kDa (Figure 4).

### 2.5. Monosaccharide Composition

**Figure 5:**
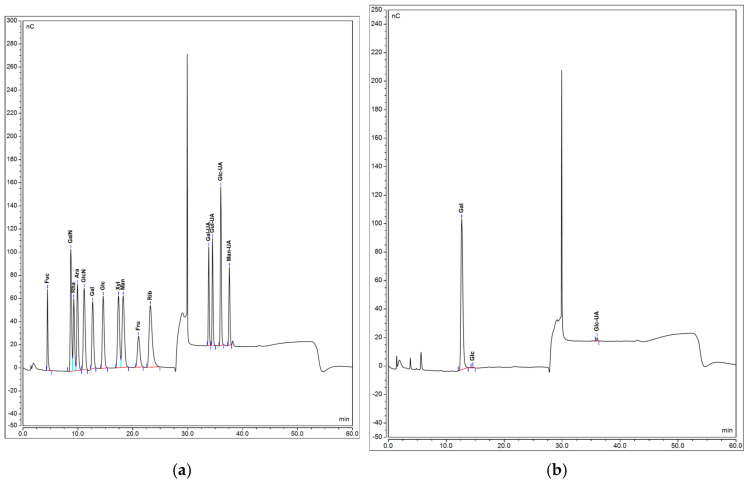
Monosaccharide composition analysis of standards (**a**) and PPHP3 (**b**).

Monosaccharide composition analysis provides fundamental compositional information for polysaccharide research and the investigation of their biological activities. As illustrated in Figure 5b, the monosaccharide components of PPHP3 include galactose (Gal), glucose (Glc), and glucuronic acid (Glc-UA), in a molar ratio of 98.3:0.46:1.24, respectively. Among these components, galactose accounts for the highest proportion, indicating that PPHP3 is a galactose-rich polysaccharide.

### 2.6. UV and FT-IR Spectral Analysis of PPHP3

**Figure 6:**
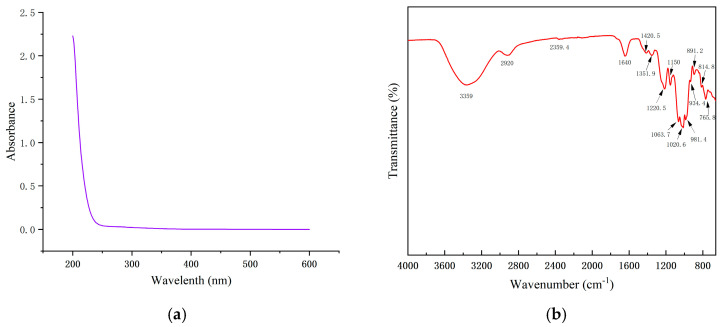
UV spectrum of PPHP3 (**a**), FT-IR spectrum of PPHP3 (**b**).

As demonstrated in Figure 6a, PPHP3 exhibits no absorption peaks at 260 nm and 280 nm, indicating that nucleic acids and proteins present in PPHP3 have been effectively removed. As shown in Figure 6b, the absorption peak detected at 3359 cm^−1^ is attributed to the stretching vibration of O–H bonds, consistent with strong hydrogen bonding within and between polysaccharide chains [26]. The absorption peak at 2920 cm^−1^ is assigned to the stretching vibration of C–H bonds [27]. Additionally, the stretching band observed at 1640 cm^−1^ corresponds to the O-H bending vibration of bound water [28]. Furthermore, the absorption peak near 1420.5 cm^−1^ is attributed to the stretching vibration of carboxyl groups (C=O) [29]. Three typical absorption peaks characteristic of pyranose rings were detected at 1150 cm^−1^, 1063.7 cm^−1^, and 1020.6 cm^−1^. Among these peaks, those at 1063.7 cm^−1^ and 1020.6 cm^−1^ correspond to the stretching vibration of ether bonds (C-O-C) and the absorption signals of hydroxyl groups in pyranose rings, respectively [30,31].

### 2.7. Scanning Electron Microscopy (SEM) Analysis

**Figure 7:**
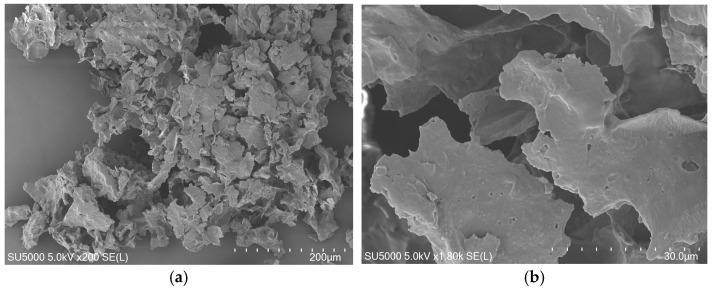
SEM images of PPHP3. (**a**) Morphology at 200× (scalebar is 200 μm); (**b**) Morphology at 1800× (scalebar is 30 μm).

As shown in Figure 7a, under 200× magnification, the SEM image of PPHP3 shows that it is composed of scattered small fragments with obvious gaps between the fragments, indicating that the particles are in a loosely aggregated state. In contrast, under 1800× magnification (Figure 7b), the surface of the flaky structure of PPHP3 is covered with small pores.

### 2.8. Hypolipidemic Activity

**Figure 8:**
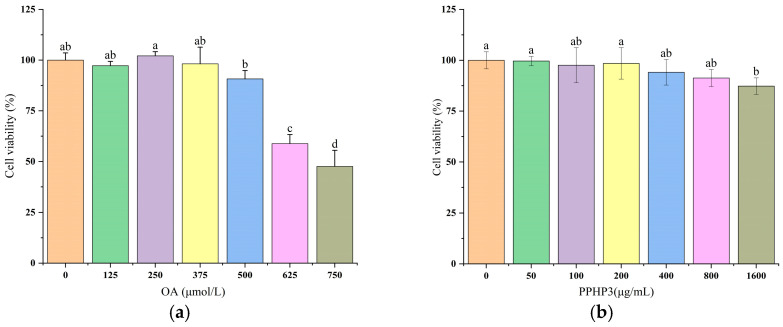

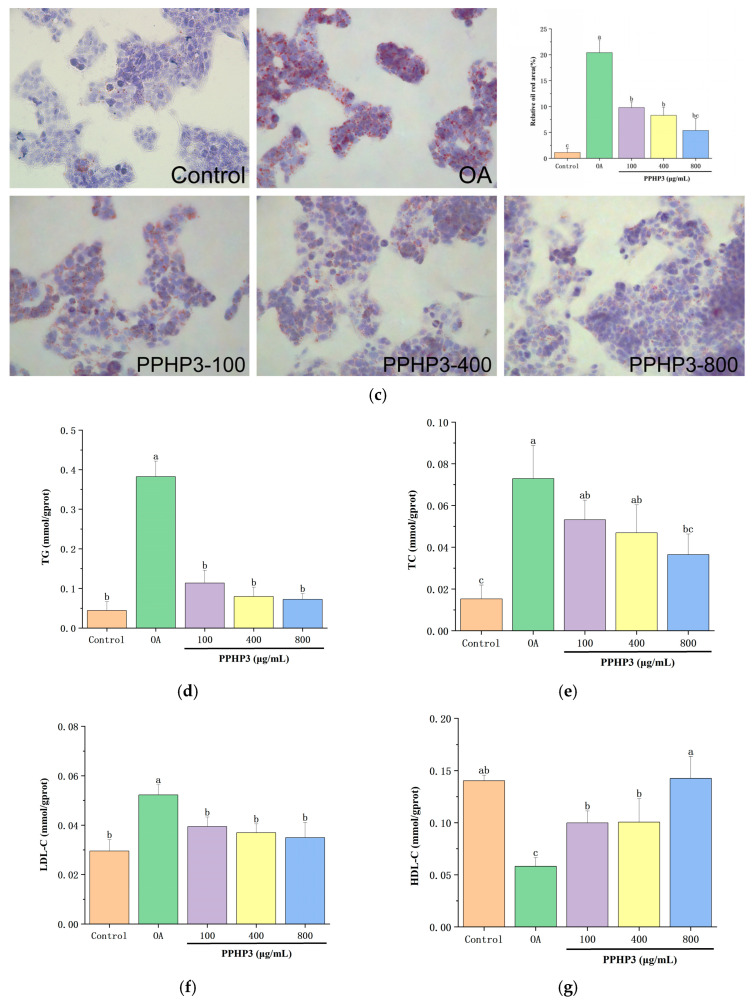
Hypolipidemic activity of PPHP3 in HepG2 cells. (**a**) Cytotoxicity of oleic acid; (**b**) Cytotoxicity of PPHP3; (**c**) Representative oil red O staining of HepG2 cells and quantification of lipid droplets accumulation; (**d**) Triglyceride (TG) content; (**e**) Total cholesterol (TC) content; (**f**) Low-density lipoprotein cholesterol (LDL-C) content; (**g**) High-density lipoprotein cholesterol (HDL-C) content. Data were shown as mean ± SD; Different letters indicate significant differences (*p* < 0.05).

The results of this study revealed that as the concentration of oleic acid gradually increased, its cytotoxicity gradually increased, significantly reducing cell viability. At a concentration of 500 μM, the cell viability was 90.75 ± 4.11%, while when the concentration increased to 625 μM, the cell viability significantly decreased to 58.85 ± 4.45%. Therefore, 500 μM OA was selected for induction modeling (Figure 8a). As illustrated in Figure 8b, PPHP3 at concentrations of 50 to 800 μg/mL had no significant effect on HepG2 cell viability, with cell survival rates maintained above 90%—a finding indicating extremely low cytotoxicity. However, the cell survival rate decreased at 1000 μg/mL PPHP3, which may be attributed to excessive osmotic pressure from the high-concentration polysaccharide solution, resulting in cell death.

Oil Red O staining results revealed that control group cells maintained intact morphology with minimal lipid deposition, whereas abundant red lipid droplets were observed in oleic acid-treated model group cells, confirming successful cell model establishment. After PPHP3 treatment, lipid droplet accumulation was significantly reduced, with the most pronounced reduction observed at 800 μg/mL, exhibiting the strongest lipid-lowering activity (Figure 8c).

Total cholesterol (TC) and triglyceride (TG) concentrations in the model group were significantly higher than those in the control group. Meanwhile, both low and high concentrations of PPHP3 reduced TG and TC levels, with the high-concentration group exhibiting particularly remarkable effects, which lowered TG and TC from 0.382 ± 0.04 mmol/g prot and 0.073 ± 0.02 mmol/g prot to 0.072 ± 0.01 mmol/g prot and 0.036 ± 0.01 mmol/g prot, respectively (Figure 8d,e). Additionally, significant differences in low-density lipoprotein cholesterol (LDL-C) and high-density lipoprotein cholesterol (HDL-C) concentrations were observed between the model and control groups. High-concentration PPHP3 significantly decreased LDL-C levels from 0.052 ± 0.004 mmol/g prot to 0.035 ± 0.006 mmol/g prot and increased HDL-C levels from 0.058 ± 0.008 mmol/g prot to 0.14 ± 0.02 mmol/g prot (Figure 8f,g).

## 3. Discussion

This study systematically investigated the extraction process, structural characteristics, and hypolipidemic activity of a polysaccharide (PPHP3) isolated from the heat-tolerant *Pyropia haitanensis* strain SW-81. Core findings revealed that the response surface methodology (RSM)-optimized ultrasonic-assisted compound enzyme extraction process achieved a high extraction rate of 10.184%, which is significantly higher than that of traditional extraction methods. Meanwhile, the homogeneous polysaccharide fraction PPHP3 isolated in this study possesses the following basic compositional features: a weight-average molecular weight (Mw) of 25.208 kDa, is composed of galactose (Gal), glucose (Glc), and glucuronic acid (Glc-UA) at a molar ratio of 98.3:0.46:1.24. These findings, together with its potent in vitro hypolipidemic activity, provide valuable references for the development of marine functional polysaccharides, both in terms of efficient extraction process methodology and bioactive component exploration.

From the perspective of process optimization, the optimized extraction rate of 10.184% achieved in this study is significantly higher than the reported value of 4.1% for *P. haitanensis* polysaccharides extracted by traditional hot water method by Shu-Ying Xu et al. [32]. Traditional extraction approaches fail to effectively disrupt the dense cell wall of *P. haitanensis*, resulting in insufficient release of bioactive polysaccharide fragments and low extraction efficiency. The ultrasonic-assisted compound enzyme method optimized via response surface methodology exhibits distinct technical advantages, relying on the synergistic effect of enzymatic hydrolysis and ultrasonic cavitation. Specifically, cellulase and pectinase selectively degrade the cellulose–pectin network of the algal cell wall, while ultrasonic cavitation strengthens mass transfer and further breaks down the cell structure to accelerate polysaccharide release. Notably, the mild extraction temperature of 65.9 °C strikes a balance between extraction efficiency and polysaccharide structural integrity, minimizing thermal degradation of glycosidic bonds. This process advantage is consistent with the findings of Reza Hashemifesharaki et al. [33], who reported that ultrasonic-assisted extraction significantly increases polysaccharide yield and enhances antioxidant activity when applied to microwave-assisted extraction of *Althaea officinalis* roots. All extraction parameters exhibited significant quadratic effects, and a strong interaction was observed between ultrasonic power and temperature, which highlights the nonlinear nature of the polysaccharide extraction process and confirms the necessity of multivariate optimization strategies [34].

In terms of structure, its moderate molecular weight is significantly lower than that of *P. haitanensis* polysaccharide PHP3 (323.8 kDa) reported by Chong Wang et al. [26] and water-extracted alcohol-precipitated *P. haitanensis* polysaccharide PHPs (630 kDa) reported by Mingshuang Dong et al. [11], but is comparable to bioactive low-molecular-weight fractions obtained via physical or chemical modification. We speculate that this difference might be attributed to the partial depolymerization of polysaccharides induced by ultrasonic treatment, which could potentially enhance their solubility and bioavailability. However, this speculation remains to be further verified by follow-up controlled experiments, to clarify the specific effect of ultrasonic treatment on the structure of polysaccharides. Monosaccharide composition analysis indicated that PPHP3 is composed of galactose (98.3%), with small amounts of glucose and glucuronic acid, indicating it is a galactose-rich polysaccharide. This result is consistent with the acidic polysaccharide characteristics of *Porphyra haitanensis* as reported by Wu et al. in prior studies [35]. In contrast, a study by Zheng et al. [36] demonstrated that the *Porphyra haitanensis* polysaccharide extracted by ultrasound was primarily composed of galactose (Gal), glucose (Glc), xylose (Xyl), mannose (Man), and galacturonic acid (GlcA), with molar ratios of 85.57 ± 0.4:2.79 ± 0.91:0.28 ± 0.4:3.24 ± 0.54:8.12 ± 1.53. These differences in monosaccharide composition are primarily attributed to variations in raw material sources and the extraction and purification methods employed in different studies.

Activity studies demonstrated that PPHP3 exhibits significant hypolipidemic activity in the oleic acid-induced HepG2 cell model. PPHP3 treatment significantly reduced intracellular triglyceride (TG) and total cholesterol (TC) levels, decreased low-density lipoprotein cholesterol (LDL-C) content and increased high-density lipoprotein cholesterol (HDL-C) content, indicating a comprehensive regulatory effect on lipid metabolism. These effects are comparable to those reported for polysaccharides from other seaweed species. Peichun Lin et al. [37] showed that three fucoidans from *Sargassum zhangii* reduced total cholesterol (TC) content in HepG2 cells without affecting cell viability in vitro. Notably, PPHP3 exhibits no cytotoxicity over a wide concentration range, further supporting its potential safety as a functional food ingredient. Zheng et al. [38] previously reported that ultra-high pressure-assisted extraction of *P. haitanensis* polysaccharides significantly reduces lipid droplets and triglyceride content in 3T3-L1 adipocytes, exhibiting significant hypolipidemic activity. The results of this study are consistent with these findings, confirming that *P. haitanensis* polysaccharides extracted by the ultrasonic-assisted compound enzyme method also exhibit hypolipidemic activity in vitro. Although the specific molecular mechanism remains to be further explored, we hypothesize that the hypolipidemic activity of PPHP3 may be mediated through multiple complementary mechanisms: first, its sulfate groups may enable bile acid binding to reduce intestinal lipid absorption, as Zheng et al. found that Bangia fusco-purpurea polysaccharides exerted hypolipidemic effects exactly via this mechanism, using their sulfate groups to bind bile acids in vitro and inhibit lipid uptake [39]; second, it may also activate the AMPK signaling pathway to modulate lipid metabolism, as the same group confirmed that this polysaccharide alleviated high-fat diet-induced dyslipidemia in mice by activating this pathway to regulate lipid metabolism [40]. These hypotheses align with established hypolipidemic mechanisms of marine polysaccharides, though they remain to be verified by further experiments.

Nonetheless, this study has certain limitations. The specific molecular mechanism underlying the hypolipidemic effect of PPHP3 remains unclarified, and in vivo validation using hyperlipidemia animal models has not been performed. Additionally, the current in vitro HepG2 cell experiments did not include a reference positive drug, which makes it difficult to benchmark the hypolipidemic efficacy of PPHP3 against established clinical agents. In addition, in-depth structural characterization (e.g., glycosidic bond sequence and chain conformation) has not been fully completed. Therefore, future research efforts will focus on three core aspects: first, evaluating the in vivo efficacy, safety, and metabolic fate of PPHP3 using hyperlipidemia animal models; second, establishing a precise structure–activity relationship via advanced techniques including NMR spectroscopy, methylation analysis, and mass spectrometry; third, exploring the underlying signaling pathways and molecular targets through transcriptomics, proteomics, and molecular docking to clarify the lipid-regulatory mechanism.

This study not only identifies PPHP3 as a promising natural hypolipidemic polysaccharide candidate but also establishes a scalable and efficient extraction process, laying a scientific basis for the development of marine polysaccharide-based functional foods and nutraceuticals.

## 4. Materials and Methods

### 4.1. Materials and Reagents

*Pyropia haitanensis* was purchased from Fujian Haisheng Co., Ltd. (Ningde, China). Cellulase (source: Trichoderma, activity ≥ 50 U/mg, product No. S10041) and pectinase (source: Aspergillus niger, activity ≥ 50,000 U/g, product No. S10007) were purchased from Shanghai Yuanye Bio-Technology Co., Ltd. (Shanghai, China). HepG2 cells were acquired from the Shanghai Cell Bank of the Chinese Academy of Sciences (Shanghai, China). Assay kits for total cholesterol (TC), triglycerides (TG), high-density lipoprotein cholesterol (HDL-c), and low-density lipoprotein cholesterol (LDL-c) were obtained from Nanjing Jiancheng Bioengineering Institute (Nanjing, China). MEM medium, fetal bovine serum, penicillin–streptomycin mixture, and PBS buffer were purchased from Gibco Life Technologies (Grand Island, NY, USA), which included all the necessary reagents for cell culture. DEAE-Sepharose FastFlow was purchased from Cytiva (Uppsala, Sweden). Sephadex-G75 was purchased from Taidu Biotechnology Co., Ltd. (Suzhou, China). Other required chemical reagents were of analytical grade.

### 4.2. Ultrasonic-Assisted Compound Enzyme Extraction

The experimental procedure described by Yu et al. [41] was adopted with slight modifications. *Pyropia haitanensis* was rinsed thoroughly and dried, then ground into powder using a grinder and sieved through a 40-mesh screen. Anhydrous ethanol was added at a ratio of 1:20 for reflux extraction to remove pigments and lipids, followed by drying and storage in sealed bags. The preprocessed *P. haitanensis* powder was blended with a compound enzyme solution at a predetermined solid–liquid ratio, homogenized, and subjected to extraction under controlled conditions of power, time, and temperature, followed by ultrasonic treatment for a certain duration. The mixture was centrifuged at 10,000 rpm for 35 min at 4 °C, and the supernatant was harvested for ethanol precipitation. The precipitate was recovered via centrifugation at 8000 rpm for 15 min at 4 °C and then redissolved. The trichloroacetic acid method was used for protein removal: trichloroacetic acid reagent and the redissolved solution were mixed at a ratio of 1:4 (*V*/*V*) and stirred for 20 min, then centrifuged to remove protein precipitate. The supernatant was subjected to dialysis and freeze-drying to yield crude polysaccharides. For the determination of total sugar content, the phenol–sulfuric acid method was adopted, with glucose used as the reference standard [42].

### 4.3. Experimental Design

#### 4.3.1. Single-Factor Experimental Design

The critical factors including liquid–solid ratio, ultrasonic treatment time, ultrasonic temperature, ultrasonic power, cellulase dosage and pectinase dosage were selected [43] to investigate the effects of extraction parameters on the total polysaccharide yield. Briefly speaking, each preprocessed powder sample was subjected to extraction at the designed solid–liquid ratios (1:10, 1:20, 1:30, 1:40, 1:50 g/mL), extraction times (30 min, 60 min, 90 min, 120 min, 150 min), extraction temperatures (50 °C, 55 °C, 60 °C, 65 °C, 70 °C), ultrasonic powers (120 W, 150 W, 180 W, 210 W, 240 W), cellulase additions (0.5%, 1.0%, 1.5%, 2.0%, 2.5% *w*/*w*), and pectinase additions (0.5%, 1.0%, 1.5%, 2.0%, 2.5% *w*/*w*). Each experiment was repeated three times.

#### 4.3.2. Response Surface Design (Box–Behnken)

On the basis of the single-factor experiments, a three-level, four-factor Box–Behnken design (BBD) was employed to assess the combined impacts of four independent variables: cellulase addition (A), pectinase addition (B), ultrasonic power (C), and ultrasonic temperature (D). The extraction rate of crude polysaccharides from *P. haitanensis* (Y%) was designated as the response variable. The detailed experimental design and corresponding extraction yields are listed in Table 1 [44].

### 4.4. Purification

For polysaccharide purification, 200 mg of the previously prepared crude polysaccharide sample was dissolved in 10 mL of distilled water, filtered to eliminate insoluble impurities, and then loaded onto a DEAE Sepharose Fast Flow column (2.6 cm × 10 cm) coupled with an AKTA purifier system. Elution was carried out with 20 mM Tris-HCl buffer, followed by sequential elution with 0 M, 0.1 M, 0.3 M, 0.5 M, 0.7 M, and 0.9 M NaCl-20 mM Tris-HCl solutions at a flow rate of 2 mL/min. The eluate was automatically collected into 10 mL fractions. The total sugar content was determined by the phenol–sulfuric acid method in a 96-well plate, with glucose used as the reference standard for quantification [45], yielding separated fractions named PHP1, PHP2, and PHP3. PHP3 was selected due to its excellent biological activity. Preliminary experiments showed that although PHP1 had a higher yield, it lacked sufficient activity, while PHP2 had a lower yield. The PHP3 fraction was subjected to desalination, concentration, and freeze-drying processes to yield polysaccharide powder. Subsequently, this polysaccharide powder was dissolved and further purified by passing through a Sephadex G-75 column (2.6 cm × 50 cm) at a flow rate of 0.5 mL/min. After collecting the eluate, the solution was concentrated and dialyzed using a 3500 Da membrane, followed by final freeze-drying to obtain the purified polysaccharide fraction PPHP3.

### 4.5. Structural Characterization

#### 4.5.1. Chemical Analysis

The total carbohydrate content was determined via the phenol–sulfuric acid assay, with glucose utilized as the calibration standard [45]. The sulfate radical content was quantified by means of the barium chloride turbidimetric method [46]. Concurrently, the BCA protein assay was adopted to measure the corresponding protein content [47].

#### 4.5.2. Molecular Weight Analysis

The absolute molecular weight and corresponding molecular weight distribution of the purified polysaccharides were characterized using a combined analytical system consisting of gel permeation chromatography, multi-angle laser light scattering and differential detector. This system consisted of a high-performance liquid chromatograph (Thermo UltiMate3000, Waltham, MA, USA), a multi-angle laser light scattering detector (Wyatt DAWN HELEOS-II, SantaBarbara, CA, USA), and a differential detector (Wyatt OPTILAB T-rex, SantaBarbara, CA, USA), which were connected in series with Ohpak SB-805 HQ and SB-803 HQ gel exclusion chromatography columns (Sizhi Technology (Hangzhou, China)) (300 × 8 mm). The prepared sample was dispersed and dissolved in 0.1 M sodium nitrate aqueous solution doped with 0.02% sodium azide, and the final concentration was adjusted to 1 mg/mL; prior to sample injection, the solution was filtered via a 0.45 μm microporous filter membrane to remove impurities. The injection volume was calibrated at 100 μL, while the mobile phase adopted the identical solvent system, along with an eluent flow velocity of 0.6 mL/min and the GPC column temperature steadily controlled at 45 °C, and isocratic elution conducted for 75 min. ASTRA 6.1 software was employed for data collection and processing, and the absolute molecular weight (Mn, Mw, Mz), polydispersity index (Mw/Mn), and root-mean-square radius of the sample were directly computed by fitting the differential signal and light scattering signal.

#### 4.5.3. Monosaccharide Composition Analysis

The monosaccharide composition of the polysaccharides was measured by ion chromatography. The specific procedures were as follows: First of all, a predefined accurate amount of the target polysaccharide specimen was accurately weighed to ensure the precision and reliability of the whole experimental manipulation. Subsequently, 1 mL of 2 M trifluoroacetic acid solution was added to the weighed sample. The resulting mixture was then transferred into a sealed tube and subjected to hydrolysis at 121 °C for a duration of 2 h. After the hydrolysis process was completed, the sample was dried by nitrogen blowing, followed by rinsing with methanol and another round of drying—this rinsing and drying cycle was repeated 2 to 3 times to thoroughly remove impurities. Finally, the obtained residue was dissolved in sterile water, and the resulting solution was filtered through a 0.22 μm filter membrane to obtain a clear sample solution for subsequent experimental testing.

All analytical determinations were performed using a Thermo ICS 5000+ ion chromatography system coupled with an electrochemical detector. The chromatographic column used was a Dionex CarboPac PA20 (Thermo Fisher Scientific, Shanghai, China) (150 × 3.0 mm, 10 μm), maintained at a column temperature of 30 °C, with an injection volume of 5 μL. The mobile phase comprised water (A), 0.1 M NaOH (B), and 0.1 M NaOH solution containing 0.2 M NaAc (C), at a flow rate of 0.5 mL/min, following a predetermined gradient elution program. Qualitative analysis was accomplished by comparing the retention times to those of 13 types of monosaccharide standards, which encompass fucose, rhamnose, arabinose, galactose, glucose, xylose, mannose, fructose, ribose, galacturonic acid, glucuronic acid, mannuronic acid, and guluronic acid. In contrast, quantitative analysis was performed by relying on the corresponding standard curves.

#### 4.5.4. UV and FT-IR Spectral Analysis

PPHP3 (1 mg/mL) was dissolved in ultrapure water. The UV absorption of PPHP3 was determined over a wavelength range of 200–600 nm using a UV spectrophotometer (UV-2600i, Shimadzu, Shanghai, China). Meanwhile, the Fourier transform infrared (FT-IR) spectrum of PPHP3 was acquired with a Thermo Nicolet iS10 Fourier transform infrared spectrometer (Thermo Inc., Waltham, MA, USA), which was equipped with a deuterated triglycine sulfate detector. All samples were tested within a wavenumber range of 600–4000 cm^−1^, with a resolution of 4 cm^−1^ and 32 cumulative scans.

#### 4.5.5. SEM Analysis

The morphology of PPHP3 was observed using a Hitachi SU 5000 scanning electron microscope (Thermo Fisher Scientific, Shanghai, China). The dried sample powder was fixed on double-sided conductive adhesive on a sample stage, sputter-coated with gold for 1.5 min, and then observed at 200× and 1800× magnifications in selected areas.

### 4.6. Hypolipidemic Activity Determination

#### 4.6.1. Cell Culture and Cell Viability Assay

HepG2 cells were propagated in MEM medium enriched with 10% fetal calf serum and 100 IU/mL penicillin–streptomycin, and incubated in a humidified cell incubator at 37 °C under an atmosphere of 95% air and 5% CO_2_, with the medium renewed every 48 h. Cell viability was assayed by the CCK-8 method: HepG2 cells were seeded into 96 well plates at a density of 5 × 10^3^ cells per well, and then exposed to different concentrations of oleic acid or PPHP3 solution for 24 h of treatment. Following treatment, 10% CCK-8 working solution was added to each individual well, followed by additional incubation in the dark for 1 h; the absorbance of each well was then detected at 450 nm. Cell viability was computed on the basis of the measured absorbance values, where As denotes the absorbance of the experimental groups and Ac denotes that of the control groups.

Cell viability (%) = (As/Ac) × 100,(2)

#### 4.6.2. Establishment of Oleic Acid-Induced Lipid Accumulation Model

HepG2 cells were seeded into 6-well culture plates at a density of 6 × 10^5^ cells per well and cultured for 24 h to facilitate cell adhesion and normal proliferation. Thereafter, the cells were exposed to 500 μM oleic acid (OA) for a further 24 h incubation to construct the cellular lipid accumulation model. Five experimental groups were designed for this study, specifically: the blank control group (cultured in MEM medium only), the model group (cultured in MEM medium supplemented with 500 μM OA), and three treatment groups (co-cultured with 100 μg/mL, 400 μg/mL, and 800 μg/mL PPHP3 respectively).

#### 4.6.3. Oil Red O Staining

After establishing the lipid accumulation model and PPHP3 intervention according to Section 4.6.2, HepG2 cells were washed three times with PBS and fixed with 4% paraformaldehyde for 20 min. The fixative was discarded, washed again with PBS, soaked in staining wash solution for 1 min, added with Oil Red O working solution for staining for 15 min, and incubated at 37 °C in the dark for 20 min. After staining, excess dye was washed off with PBS, and cell images were collected under an upright microscope. After image collection, quantitative analysis was performed using Image-J 1.53t software.

Oil Red O relative area (%) = (Oil Red O area/Cell area) × 100,(3)

#### 4.6.4. Lipid Content Determination

Following the construction of the cellular lipid accumulation model and the implementation of PPHP3 intervention, the intracellular levels of triglycerides, total cholesterol, high-density lipoprotein cholesterol as well as low-density lipoprotein cholesterol were quantified via commercial assay kits, and all experimental outcomes were presented in units of millimoles per gram of protein.

### 4.7. Statistical Analysis

All experiments were conducted in at least three independent parallel experiments, and the data were presented as mean ± standard deviation. Data samples were collated by Excel 2016 software, plotted using OriginPro 2021 software, and analyzed by IBM SPSS Statistics 27.0 software using Tukey’s method for multiple comparisons with *p* < 0.05 being a statistically significant difference.

## 5. Conclusions

This study systematically optimized the extraction process of crude polysaccharides from the thermotolerant *Pyropia haitanensis* strain SW-81 using single-factor screening combined with response surface methodology (RSM). The optimal extraction parameters were determined as follows: liquid–solid ratio of 30 mL/g, 1.48% cellulase, 1.47% pectinase, 180 W ultrasonic power, 65.9 °C extraction temperature and 90 min ultrasonic time, under which the polysaccharide yield reached 10.184%. The purified homogeneous polysaccharide fraction PPHP3 was successfully isolated. Basic compositional analysis showed that PPHP3 had a molecular weight of 25.208 kDa and was mainly composed of galactose, glucose and glucuronic acid at a molar ratio of 98.3:0.46:1.24. In vitro bioactivity assays verified that PPHP3 presents significant hypolipidemic effects in oleic acid-induced HepG2 cells with low cytotoxicity. These results provide a solid scientific foundation for the development and application of *P. haitanensis* polysaccharides in lipid-lowering functional foods and marine bioactive products. Further studies will be carried out to verify their in vivo activity. Notably, detailed structural analysis of this polysaccharide, including glycosidic linkage types, sulfate group position and distribution, and 3,6-anhydrogalactose content, is currently ongoing and will be reported separately, which will help to fully elucidate the structure–activity relationship and further promote its application in functional food products.

## Figures and Tables

**Table 1:** The response surface design and extraction yield of polysaccharides.

Run	(A) Cellulase Addition (%)	(B) Pectinase Addition (%)	(C) Ultrasonic Power (W)	(D) Ultrasonic Temperature (°C)	Extraction Yield (%)
1	1.5	1.5	150	70	9.186
2	2	1.5	210	65	7.849
3	1.5	1	180	60	8.369
4	1.5	1	180	70	8.537
5	1	1.5	180	60	7.854
6	1.5	2	210	65	7.963
7	2	1.5	180	70	7.749
8	1.5	1.5	150	60	6.584
9	1.5	1.5	180	65	10.257
10	1	1.5	150	65	7.856
11	1.5	1.5	180	65	9.981
12	2	1.5	180	60	7.416
13	1.5	1.5	180	65	10.871
14	1.5	1.5	180	65	10.276
15	2	1.5	150	65	7.911
16	1.5	1.5	180	65	10.116
17	1.5	1.5	210	60	7.929
18	1.5	2	180	60	7.788
19	2	1	180	65	7.859
20	1	1	180	65	8.393
21	1.5	2	180	70	8.69
22	1.5	1.5	210	70	8.384
23	1	1.5	180	70	8.113
24	1.5	1	150	65	8.552
25	2	2	180	65	7.945
26	1.5	1	210	65	8.228
27	1	2	180	65	7.827
28	1	1.5	210	65	7.911
29	1.5	2	150	65	8.06

**Table 2:** Analysis of variance for a fitted regression equation of the extraction yield of *P. haitanensis* polysaccharides.

Source	Sum of Squares	Degree of Freedom	Mean Square	F-Value	*p*-Value	Significance
Model	25.55	14	1.82	15.99	<0.0001	**
A-cellulase addition	0.1251	1	0.1251	1.1	0.313	
B-Pectinase addition	0.231	1	0.231	2.02	0.1767	
C-Ultrasonic power	0.0011	1	0.0011	0.0097	0.9231	
D-Ultrasonic temperature	1.86	1	1.86	16.26	0.0012	**
AB	0.1063	1	0.1063	0.9311	0.3509	
AC	0.0034	1	0.0034	0.03	0.865	
AD	0.0014	1	0.0014	0.012	0.9143	
BC	0.0129	1	0.0129	0.1129	0.7419	
BD	0.1347	1	0.1347	1.18	0.2957	
CD	1.15	1	1.15	10.1	0.0067	**
A^2^	11.9	1	11.9	104.25	<0.0001	**
B^2^	5.41	1	5.41	47.42	<0.0001	**
C^2^	8.4	1	8.4	73.62	<0.0001	**
D^2^	8.06	1	8.06	70.64	<0.0001	**
Residual	1.6	14	0.1141			
Lack of Fit	1.13	10	0.1134	0.9773	0.5589	not significant
Pure Error	0.4641	4	0.116			
Cor Total	27.15	28				

** highly significant (*p* < 0.01), not significant (*p* > 0.05).

**Table 3:** Chemical composition of PPHP3.

Component	PPHP3
Total sugar (%)	84.34 ± 2.25
Protein (%)	0.89 ± 0.05
Sulfate content (%)	8.64 ± 0.05
Molecular weight (kDa)	25.208

## Data Availability

The data presented in this study are available on request from the corresponding author.
